# Social support during pregnancy and the risk of postpartum depression in Polish women: A prospective study

**DOI:** 10.1038/s41598-024-57477-1

**Published:** 2024-03-22

**Authors:** Joanna Żyrek, Magdalena Klimek, Anna Apanasewicz, Aleksandra Ciochoń, Dariusz P. Danel, Urszula M. Marcinkowska, Magdalena Mijas, Anna Ziomkiewicz, Andrzej Galbarczyk

**Affiliations:** 1 Department of Human Behavior, Ecology and Culture, Max Planck Institute for Evolutionary Anthropology, Leipzig, Germany; 2 Department of Environmental Health, Institute of Public Health, Faculty of Health Sciences, Jagiellonian University Medical College, Krakow, Poland; 3 Department of Anthropology, Ludwik Hirszfeld Institute of Immunology and Experimental Therapy, Polish Academy of Sciences, Wroclaw, Poland; 4 Doctoral School of Medical and Health Sciences, Jagiellonian University Medical College, Krakow, Poland; 5 Laboratory of Anthropology, Institute of Zoology and Biomedical Research, Jagiellonian University, Krakow, Poland; 6 BirthRites Lise Meitner Research Group, Max Planck Institute for Evolutionary Anthropology, Leipzig, Germany

**Keywords:** COVID-19, Perceived support, Postpartum depression, Psychological care, Well-being, Risk factors, Human behaviour

## Abstract

Social support has been proposed as an important determinant of women's physical and emotional well-being during pregnancy and after childbirth. Our study aimed to examine the association between the risk of postpartum depression (PPD) and perceived social support during pregnancy. A web-based prospective study survey was conducted among Polish women. The level of social support was measured with the Berlin Social Support Scales during pregnancy. Four weeks after the birth the risk of PPD was assessed using the Edinburgh Postpartum Depression Scale. Data from 932 mothers aged 19–43 (mean 30.95; SD 3.83) were analyzed using multinomial logistic regression. Higher perceived available support (emotional and instrumental), currently received support (emotional, instrumental and informational), satisfaction with the support, and sum of score were all associated with lower risk of PPD, after controlling for selected covariates (woman's age, socioeconomic status, parity status, place of residency, education, child's Apgar score, type of delivery, complications during birth, kin assisting the labor, breastfeeding). Our results suggest that the more social support the pregnant woman receives, the lower is her risk of PPD. Since humans evolved as cooperative breeders, they are inherently reliant on social support to raise children and such allomaternal help could improve maternal well-being.

## Introduction

Postpartum depression (PPD) is a mental health condition associated with pregnancy and childbirth. According to the DSM-5, it is defined as symptoms occurring within four weeks after delivery that meet the criteria for a major depressive disorder (MDD) episode^1^. World Health Organization (WHO) estimates that about 13% of postpartum women experience this health adversity, with the caveat that the percentage is higher in economically developing countries^1–3^. In response to the need for updated data on the global prevalence of PPD, the most comprehensive systematic review of the literature with a meta-analysis was conducted in 2021^4^. The analysis included 565 studies from 80 different countries and world regions with a total sample of 1,236,365 women. The results indicated that the global prevalence of PPD was about 17.22% (95% CI 16.00–18.51)—a higher percentage than the one estimated by the WHO. As of today, there are no population-based epidemiological studies or registries in Poland that provide information on the prevalence of PPD^2^. However, starting from 2019, a project called “Next Stop: Mum” is being implemented in Poland as part of the National Health Policy Program, which provides unequivocal information on the prevalence of PPD. Results from three northern provinces (voivodships in the Polish nomenclature of administrative division) where the survey was conducted showed that when assessed by midwives, the prevalence of PPD was about 7%. However, a telephone survey subsequently conducted to monitor the effects of the project indicated that the incidence of PPD was approximately 12%^5^. Meanwhile, on the online platform of the program, where a test based on the Edinburgh Postpartum Depression Scale (EPDS) is available, results showed that PPD was indicated in 70% of the 12,000 women who completed it^5^.

PPD affects the relationship between mother and child, leading to difficulties in bonding, emotional attachment, breastfeeding, and the mother’s difficulties in addressing her child’s needs^6^. In addition, studies indicate that PPD causes suffering for the mother, worsens her well-being, and increases the risk of marital conflict^1^. Moreover, infants of mothers suffering from PDD show decreased activity, intensified crying, nervousness, or disturbances in sleep and wakefulness^6^. Maternal PPD may also have a long-term effect on a child's mental health problems^7^. Older children of mothers affected by PPD may exhibit anxiety, depressive and aggressive behaviors, and hyperactivity to stress^6,8,9^.

Social support is a multidimensional and complex concept that has been studied for many years to define it and develop tools to measure it^10^. It can be viewed in the form of instrumental support (e.g., tangible actions such as shopping groceries), emotional support (e.g., providing encouragement, reassurance, compassion), advice or information (exchange of information that facilitates understanding of the situation and helps to solve the problem), financial support, provision of care, moral support, and social ties with others^10–12^. This complex support is an important determinant of women's physical and emotional well-being during pregnancy and after childbirth^13,14^, and can have a positive impact on making beneficial choices, such as breastfeeding^15^. It has been suggested that social support, particularly its emotional dimension, represents a protective factor against parenting stress and is a significant predictor of PPD^16^. Thus, a low level of support is identified as one of the most significant psychosocial risk factors for the development of PPD^17,18^. Previous studies suggest that there is a significant negative association between social support during and after pregnancy and PPD, but these should be evaluated in light of certain limitations, such as relatively small study groups^17,19,20^. This implies the need for further prospective studies to examine the impact of social support on the development of PPD and to assess in more detail which dimensions of social support benefit women the most.

From an evolutionary perspective, it is important to point out that humans evolved as cooperative (communal) breeders^21,22^. Thus, relying on allomaternal help and exhibiting collective childrearing behaviors is not surprising^18,23^. Because of that, mothers have an innate need to involve their social network in raising and investing in their offspring. Consequently, one could expect that in case of complete absence or a very low social support, mothers may perceive childcare as excessively difficult, which in turn may lead to an increased risk of PPD.

Due to the outbreak of a pandemic caused by the SARS-CoV-2 virus in 2020, the topic of the impact of social support and social isolation on human health has become particularly important. This situation has dramatically reduced access to and support from broad social networks, instilled fear in the public and increased stress levels^24^. Women who gave birth during the pandemic were particularly affected—in many cases, they did not receive social support and assistance in caring for their newborns, which, along with isolation and anxiety, may have contributed to increased depressive symptoms^18,24^. The pandemic and subsequent social distance made perinatal support less available, increasing adverse postpartum mental health outcomes^24^. Such connections have gradually been demonstrated in studies conducted during the pandemic, with results from the UK and the US showing an increase in the incidence of PPD and other mental health issues compared to the pre-pandemic period^18,24^. Additionally, the available virtual support seems to have not proven effective in addressing the issue (i.e., women participating in online antenatal classes had similar levels of anxiety and depression as those who did not attend any form of classes—in comparison to stationary meetings)^25^.

This study examined the association between social support during pregnancy and the risk of PPD in Polish women. Participants in our study were in their second or third trimester of pregnancy at the time of study recruitment, which was carried out during the pandemic caused by the SARS-CoV-2 virus (COVID-19). In particular, we focused on the association with PPD of such dimensions of social support as perceived available support (emotional, instrumental), need for support, seeking support, currently received support (emotional, instrumental, informational), and satisfaction with support. We hypothesized that social support during pregnancy is a protective factor against PPD, and therefore women with higher levels of various dimensions of social support should demonstrate a reduction in depressive symptoms, leading to a diminished risk of PPD.

## Methods

### Study population

**Figure 1:**
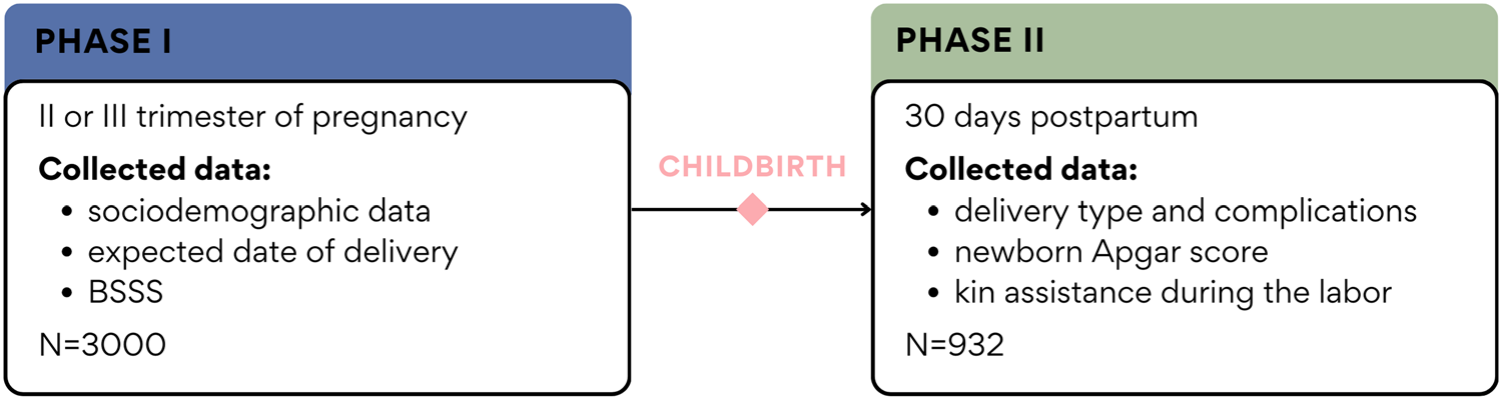
Overview of the study design.

Our analysis used data from the “Corona Mums” prospective study (https://osf.io/5cveq/), which aimed to investigate the relationship between perceived levels of pregnancy stress and anxiety during the COVID-19 pandemic and the physical and psychological well-being of both the pregnant woman and the newborn (see also: Ref.^25^). The project lasted from May 2020 to September 2021. The first stage included an online survey targeting pregnant women, in which more than 3,000 women participated. They were invited to the study through local and national newspapers and radio stations, social media (Facebook), and community forums for pregnant women. The second phase of the survey began in November 2021 and targeted women after childbirth who had previously (during the first wave of the survey) agreed to participate in the subsequent phase of the study. The second questionnaire was sent to women two weeks after the date of expected delivery, which was indicated during the first survey. A total of 932 women completed both phases of the study and were included in the present analyses. The study was performed in accordance with the Declaration of Helsinki. The study protocol was approved by the Jagiellonian University Bioethics Committee (date: 28/05/2020, decision number: 1072.6120.141.2020). Informed consent was obtained from all participants. Figure 1 provides an overview of the study design.

### Questionnaires

During the first phase of the study, pregnant women completed a survey collecting sociodemographic data (e.g., age, education, place of residency) and information about the course of pregnancy (e.g., expected date of delivery). They were also asked to assess their social support. The assessment of social support during pregnancy was based on the Berlin Social Support Scales (BSSS) developed by Schwarzer and Schwarzer^26^; Polish adaptation and validation by Łuczyńska et al.^27^. The original questionnaire includes questions about support from relatives and users of online forums. However, in our analysis, we used only the items about relatives because many of our participants declared that they were unwilling to look for social support online. The BSSS includes the following scales and subscales: perceived available support (emotional and instrumental), need for support, support seeking, currently received support (emotional, instrumental, informational, and satisfaction with support), and buffer-protective support. The different scales can be used separately or together, and by adding up the scales of perceived available support and currently received support, a sum of support can be obtained. This is a self-report measurement in which the respondent rates compliance with a statement on a 4-point scale. The results of the Polish adaptation of the BSSS indicated that it is a consistent and accurate measure of social support^27^.

During the second phase, we additionally collected information about the course of delivery and the child's health status (e.g., delivery type and complications, newborn Apgar score, and kin assistance during the labor). PPD was evaluated after birth using the EPDS developed by Cox et al.^28^ and adapted and validated for the Polish population by Kossakowska ^29^. It is a 10-item self-report screening tool with a 4-point Likert scale ranging from 0 to 3 for each question and a maximum score of 30. A higher score represents a higher risk of PPD. A score of 12/13 is often taken as the cut-off value for developing PPD, as determined in both the original and validation studies^30^. The Polish version of EPDS was assessed to have good psychometric properties^29^.

### Statistical analysis

Logistic regression (logit models) was used to determine the relationship between dimensions of social support (BSSS score) and the incidence of PPD (EPDS score). Each dimension of social support was analyzed in a separate logistic regression model. We divided our sample according to EPDS score (cut-off value 12/13 points) into groups without and with depression. Participants with a score of at least 13 were classified as having PPD.

The control variables were selected based on previously defined risk factors for PPD^31–34^ and included: mother's age, current socioeconomic status (SES; subjective assessment on a scale of 1–7), baby's Apgar score, education (tertiary vs. lower than tertiary), pregnancy complications (yes/no), previous deliveries (primiparous/multiparous), place of residence [large city (over 100,000 population)/smaller town (less than 100,000 population)], breastfeeding (yes/no), kin assisting the labor (yes/no), and type of delivery (vaginal/cesarean section). Each model included the same set of control variables. A probability value of p < 0.05 represented statistically significant results. The analyses were performed in Statistica version 13.0.

## Results

The study group included 932 women from Poland, aged 19–43 (mean 30.95; SD 3.83). None of the study participants smoked cigarettes or drank alcohol during pregnancy. At the time of recruitment to the study, women were in the second or third trimester of pregnancy during the COVID-19 pandemic. Even though the survey was sent two weeks after the predicted delivery, the women filled it out approximately four weeks after labor (mean 30 days; SD 18.08). Most participants (56.97%) came from large cities (more than 100,000 inhabitants) and acquired higher education (at least a bachelor’s degree; 84.55%). Of the 932 women in the study, 166 (17.81%) had PPD according to EPDS criteria; on average, women scored 7.6 points (SD 5.68). The characteristics of the study participants and the questionnaire scores are presented in Table 1.

**Table 1:** The characteristics of study participants and the questionnaire scores among all women, women with and without PPD.

	All women (N = 932)		Without PPD (N = 766)		With PPD (N = 166)		t	p
	Mean	SD	Mean	SD	Mean	SD		
Age [years]	30.95	3.83	31.10	3.79	30.25	3.92	− 2.62	**0.009**
Socioeconomic status (SES) [score]	6.35	1.26	6.40	1.23	6.10	1.34	− 2.82	**0.005**
EPDS score	7.60	5.68	5.57	3.58	17.02	3.71	37.17	**< 0.001**
Apgar score	9.74	0.90	9.80	0.70	9.50	1.49	− 4.52	** < 0.001**
Dimensions of social support [scores]								
Sum of support	76.65	10.23	77.36	9.65	73.38	12.07	− 4.58	** < 0.001**
Perceived available emotional support	13.81	1.96	13.92	1.89	13.29	2.18	− 3.76	** < 0.001**
Perceived available instrumental support	14.32	1.99	14.45	1.86	13.72	2.41	− 4.35	** < 0.001**
Need for support	11.83	2.29	11.75	2.26	12.20	2.37	2.29	**0.022**
Support seeking	14.25	2.63	14.23	2.60	14.31	2.74	0.33	0.743
Currently received emotional support	31.62	4.96	31.92	4.64	30.25	6.02	− 3.98	** < 0.001**
Currently received instrumental support	10.51	1.75	10.61	1.65	10.02	2.08	− 4.01	** < 0.001**
Currently received informational support	6.39	1.51	6.45	1.47	6.02	1.67	− 2.65	**0.01**

	n	%	n	%	n	%	Χ^2^	P
Parity status							11.48	** < 0.001**
Primiparous	530	56.87	350	45.69	52	31.33		
Multiparous	402	43.13	416	54.31	114	68.67		
Type of delivery							2.34	0.126
Vaginal	571	61.27	478	62.40	93	56.02		
Caesarean section	361	38.73	288	37.60	73	43.98		
Pregnancy complications							0.095	0.758
Yes	341	36.59	282	36.81	59	35.54		
No	591	63.41	484	63.19	107	64.46		
Kin assisting the labor							2.35	0.125
Yes	516	55.36	433	56.53	83	50.00		
No	416	44.64	333	43.47	83	50.00		
Breastfeeding							7.43	**0.006**
Yes	835	89.59	696	90.86	139	83.73		
No	97	10.41	70	9.14	27	16.27		
Place of residence							0.18	0.675
City > 100.000 residents	531	56.97	434	56.66	97	58.43		
City < 100.000 residents	401	43.03	332	43.34	69	41.57		
Education							0.10	0.749
Tertiary	788	84.55	649	84.73	139	83.73		
Lower than tertiary	144	15.45	117	15.27	27	16.27		
Satisfaction with support							35.62	** < 0.001**
1 (strongly disagree)	16	1.72	10	1.31	6	3.61		
2 (somewhat disagree)	63	6.76	36	4.70	27	16.27		
3 (somewhat agree)	206	22.10	168	21.93	38	22.89		
4 (strongly agree)	647	69.42	552	72.06	95	57.23		

Statistically significant differences are bolded.

**Figure 2:**
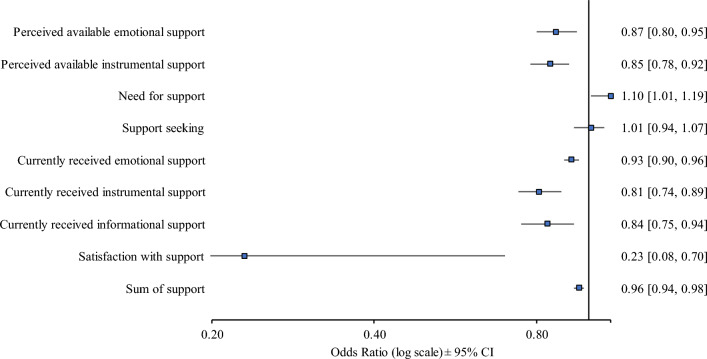
Forest plot presenting the result of multivariate logistic regressions for postpartum depression. The plot presents the likelihood of postpartum depression in relation to individual dimensions of social support and sum of social support. Odds ratios and 95% confidence intervals (CI) are depicted. Significant associations are shown when CIs do not overlap with 0.

The results obtained from logistic regression models indicate that a lower risk of PPD was associated with higher levels of most dimensions of social support, as measured by the BSSS. Lower risk of PPD was related to higher perceived available support (emotional [OR = 0.87, 95% CI 0.80–0.95, p = 0.001], instrumental [OR = 0.85, 95% CI 0.78–0.92, p < 0.001]), currently received support (emotional [OR = 0.93, 95% CI 0.90–0.96, p < 0.001], instrumental [OR = 0.81, 95% CI 0.74–0.89, p < 0.001], informational [OR = 0.84, 95% CI 0.75–0.94, p = 0.002]), satisfaction with support (OR = 0.23, 95% CI 0.08–0.70, p = 0.004), and sum of score (OR = 0.96, 95% CI 0.94–0.98, p < 0.001). In turn, a higher risk of PPD was related to a higher need for support (OR = 1.10, 95% CI 1.01–1.19, p = 0.024). No statistically significant relationship was observed between the risk of PPD and support-seeking dimensions (OR = 1.01, 95% CI 0.94–1.07, p = 0.875). All models were controlled for the mother's age, current SES, baby's Apgar score, education, pregnancy complications, parity status, place of residence, breastfeeding, kin assisting the labor, and type of delivery (Fig. 2; Supplementary Table 1) . Of the control variables, Apgar scores, multiparity, and current SES had statistically significant effects on the risk of PPD in most models—higher Apgar scores, previous birth, and higher SES lowered the risk of PPD.

## Discussion

Based on the results of our study, 17.81% of women could be classified as suffering from PPD (with accordance to the cutoff point of 12/13 EPDS). This is in line with the results obtained in the largest systematic literature review to date, where the global prevalence of PPD was estimated to be around 17.22%^4^. However, in the Polish context, our results showed a higher prevalence of PPD when compared with the results of the ongoing project “Next Stop: Mum” (7% when assessed by midwives and 12% during a telephone survey). Interestingly, that project also included an online platform and the results showed that PPD was indicated in 70% of the women who completed it. Nonetheless, when comparing data on the prevalence of PPD, we should consider the time and the method of data collection, as well as the studied population. When it comes to the time of data collection, in our study, the EPDS-based test was completed after an average of 30 days (SD 18.08) postpartum, while in “Next Stop: Mum” data was collected up to a year after delivery. Moreover, we used an online survey as a data collection method that limits direct contact (compared to data collection by midwives or a telephone survey), and thus may be a more reliable source of information, since, given the sensitivity of the issue, women may be less prone to directly share their feelings, concerns, and struggles for fear of being judged. Also, the online survey can give them a greater sense of anonymity. Given that the “Next Stop: Mum” project also collected data via an online survey, it should be noted that the authors themselves recommend caution in interpreting the indicated high prevalence of PPD. This is because the public online platform is mainly the source of help and information for individuals who may already be experiencing depressive symptoms, and therefore the data should not be considered as representative and generalized on the population of postpartum women^5,35^.

In general, our results underline the significant role of social support during pregnancy in reducing the risk of developing PPD independently of the numerous control variables, such as pregnancy complications, education, place of residence, breastfeeding, or type of delivery. We tested nine social support subscales from the BSSS, and for seven of them (with the exception of support-seeking), we confirmed our hypothesis that the higher levels of social support during pregnancy were significantly associated with a lower risk of PPD. In turn, the higher score of the need for support dimension was significantly associated with a higher risk of PPD. This applies to both perceived (subjective perceptions of the support available from others) and received (actually received, i.e., specific supportive activities) support, which are considered separate constructs that can demonstrate different relationships with variables of interest^36^. The strongest association was found for currently received instrumental and informational support. Unsurprisingly, higher socioeconomic status^37^ and Apgar scores were associated with a lower risk of PPD^38,39^. This study also confirmed the significant role of multiparity for PPD—those women for whom this was not their first birth had a lower risk of PPD than women who had never given birth before^40^.

To the best of our knowledge, this is the second overall and the first prospective study assessing the relationship between social support and the risk of PPD conducted among women living in Poland. The first study, using similar measurement methods (i.e., the EPDS scale with a cut-off point taken at 13 points, the BSSS, and regression analysis) was conducted in 2012 on a much smaller sample of 101 women who had given birth at the Department of Obstetrics of the Medical University of Gdansk^19^. Nevertheless, the profile of the study participants was similar—they were mostly women from large cities and with tertiary education, whose mean EPDS score was 7.92. In that study, the authors showed that dimensions of social support, such as perceived available instrumental support and need for support, had a significant impact on the risk of PPD. These results are only partially consistent with ours, as the directions of the relationship are the same, but the magnitude of the effect is smaller. It is also important to emphasize that in that study, social support was assessed after pregnancy, unlike in our study, where it was measured during pregnancy^19^. The observed differences in results compared to our study may be related to methodological differences, including study design, accounting for different confounding factors, and a smaller sample size^19^.

Considering PPD from an evolutionary perspective, it is necessary to refer to the fact that humans evolved as cooperative (communal) breeders—they rely on allomaternal help to care for their children and tend to exhibit cooperative childrearing behaviors in order to increase the offspring’s survival probability^18,23^. This emphasizes the need to involve members of the mother’s social network in raising and investing in the offspring. The evolutionary perspective, in a way, relieves the woman of complete responsibility for the childcare burden and shows the need for relatives and society to provide assistance to ensure the child’s proper development and the woman’s health^23^. As cooperative breeders, humans need extensive support, both during pregnancy and after childbirth (during the process of raising children) due to the significant energy costs of parental care. The lack of support leads to numerous mental health adversities for mothers, such as PPD, anxiety, and stress, which might result in decreased investment in the child^41,42^. In our study, we explored the effect of general social support, but a recent study suggests that it is important from whom this support is received, for example, whether from the husband or from other family members^18^. Therefore, to achieve a more comprehensive understanding of the impact of social support, future research should also consider such connections.

Lack of family or social support is a major problem associated with relatively recent socioeconomic changes, in particular, the transition from multigenerational to nuclear families. Nowadays, often it is the father who remains the only relative providing key support to the mother in raising the children^18^. However, it is common that a woman who cannot count on sufficient support from kins, seeks and finds support from other sources. As we demonstrated previously, during the COVID-19 pandemic, women who attended inpatient antenatal classes had significantly lower levels of anxiety (as measured by the STAI-State tool) compared to women who did not participate in such classes^25^. In addition, the same trend was observed for the occurrence of depression during pregnancy—women who attended the antenatal classes had a significantly lower score on the EPDS compared to women who did not participate^25^. Therefore, it could be expected that, in addition to educational function, antenatal classes also play a role in the psychosocial well-being of the pregnant woman. They provide an opportunity to obtain support at all its dimensions, mainly emotional and informational, through the exchange of information and experiences between parents-to-be so they can better prepare themselves for the upcoming changes. Moreover, a woman attending the birthing classes can expand her social network increasing received social support.

Although the strengths of our study are its sample size (932 women) and its prospective design, our results should be interpreted in light of certain limitations. First, no data were collected on pre-existing psychiatric conditions, such as depression or bipolar disorder, which are strongly associated with the risk of PPD^31^. Second, we have assessed PPD after an average of four weeks postpartum, however it is established that PPD can occur within 6 weeks after childbirth to even the second half of a child's first year and given that our results may be underestimated. Third, the study sample was relatively homogenous—most women were from large cities, highly educated, and possessing high socioeconomic status. Fourth, our study relied on self-reported data collected through an online platform. It is possible then that participants’ ratings related to PPD symptoms could be underestimated or overestimated depending on individual characteristics^43^. Fifth, we collected data during the COVID-19 pandemic, and for this reason, our results should be considered with caution, as generalizing to another period may be difficult.

In summary, the results from this study demonstrate the crucial role of various kinds of support during pregnancy for maternal well-being, with a particular focus on their mental health. Pregnant women who receive greater support and are more satisfied with the level of support are less likely to develop PPD. These findings also highlight the need to identify women with low levels of support, as well as those with other risk factors, such as low socioeconomic status, younger age, or high-risk pregnancy status, due to the fact that they are more likely to experience depressive symptoms after delivery^6,31^.

Finally, our results can support the development of effective interventions focused on women’s emotional support in order to decrease risk of PPD. One such intervention could be education of pregnant women and their partners during antenatal classes focused on the importance of the role of loved ones’ support in minimizing postpartum complications and the occurrence of PPD.

### Supplementary Information

Supplementary Information.

## Data Availability

The datasets used and analyzed during the current study are available from the corresponding author on reasonable request.
