# Supplementary material for: Social support during pregnancy and the risk of postpartum depression in Polish women: A prospective study
Source: Sci Rep. 2024 Mar 22;14:6906. doi: 10.1038/s41598-024-57477-1 (PMC10959954; doi:10.1038/s41598-024-57477-1)
Supplement: Supplementary file 1 — Supplementary Information. [file 41598_2024_57477_MOESM1_ESM.pdf]

Social support during pregnancy and the risk of postpartum depression in Polish women. A prospective study.

Joanna Żyrek, Magdalena Klimek, Anna Apanasewicz, Aleksandra Ciochoń, Dariusz P. Danel, Urszula M. Marcinkowska, Magdalena Mijas, Anna Ziomkiewicz, Andrzej Galbarczyk

Supplementary Table 1 *The likelihood of postpartum depression in relation to social support dimensions, after including control variables in the models. Results in bold are statistically significant (p<0.05).*

	Model 1			Model 2			Model 3			Model 4			Model 5			Model 6			Model 7			Model 8			Model 9		
	OR	95%CI	p	OR	95%CI	p	OR	95%CI	P	OR	95%CI	p	OR	95%CI	p	OR	95%CI	p	OR	95%CI	p	OR	95%CI	p	OR	95%CI	p
Perceived available emotional support	**0.87**	**0.80;0.95**	**0.001**																								
Perceived available instrumental support				**0.85**	**0.78;0.92**	**<0.001**																					
Need for support							**1.10**	**1.01;1.19**	**0.024**																		
Support seeking										1.01	0.94;1.07	0.875															
Currently received emotional support													**0.93**	**0.90;0.96**	**<0.001**												
Currently received instrumental support																**0.81**	**0.74;0.89**	**<0.001**									
Currently received informational support																			**0.84**	**0.75;0.94**	**0.002**						
Satisfaction with support																						**0.23**	**0.08;0.70**	**0.004**			
Sum of support																									**0.96**	**0.94;0.98**	**<0.001**
Age	0.97	0.92;1.02	0.221	0.97	0.92;1.02	0.480	0.98	0.93;1.03	0.480	0.97	0.93;1.02	0.875	0.96	0.92;1.01	0.162	0.96	0.92;1.01	0.157	0.97	0.92;1.01	0.166	0.97	0.92;1.02	0.256	0.96	0.92;1.01	0.152
Socioeconomic status (SES)	**0.86**	**0.74;0.99**	**0.039**	**0.87**	**0.76;1.01**	**0.005**	**0.82**	**0.71;0.94**	**0.005**	**0.82**	**0.71;0.95**	**0.286**	**0.86**	**0.75;1.00**	**0.049**	**0.86**	**0.75;1.00**	**0.043**	**0.85**	**0.74;0.98**	**0.028**	0.90	0.77;1.05	0.166	0.88	0.76;1.02	0.099
Apgar score	**0.76**	**0.65;0.90**	**0.001**	**0.77**	**0.65;0.9**	**<0.001**	**0.75**	**0.64;0.88**	**<0.001**	**0.75**	**0.64;0.88**	**0.006**	**0.75**	**0.64;0.88**	**<0.001**	**0.74**	**0.63;0.87**	**<0.001**	**0.75**	**0.64;0.88**	**<0.001**	**0.75**	**0.63;0.90**	**0.002**	**0.76**	**0.64;0.89**	**0.001**
Education (Tertiary)	1.09	0.67;1.79	0.721	1.14	0.69;1.87	0.776	1.07	0.66;1.75	0.776	1.09	0.67;1.77	<0.001	1.06	0.64;1.73	0.831	1.07	0.65;1.76	0.780	1.08	0.66;1.77	0.746	1.04	0.61;1.78	0.885	1.08	0.65;1.77	0.773
Pregnancy complications (No)	1.01	0.70;1.45	0.963	1.00	0.70;1.45	0.982	1.00	0.70;1.45	0.982	1.00	0.70;1.44	0.742	1.05	0.73;1.52	0.801	1.01	0.70;1.46	0.948	1.00	0.70;1.45	0.987	1.25	0.83;1.88	0.284	1.04	0.72;1.50	0.852
Parity status (Multiparous)	**0.53**	**0.35;0.79**	**0.002**	**0.52**	**0.34;0.77**	**0.003**	**0.55**	**0.37;0.82**	**0.003**	**0.57**	**0.38;0.84**	**0.998**	**0.48**	**0.31;0.72**	**<0.001**	**0.50**	**0.33;0.75**	**0.001**	**0.52**	**0.35;0.78**	**0.002**	**0.48**	**0.31;0.75**	**0.001**	**0.47**	**0.31;0.71**	**<0.001**
Place of residence (City over 100,000 residents)	1.12	0.77;1.61	0.550	1.08	0.75;1.56	0.552	1.12	0.78;1.61	0.552	1.12	0.78;1.62	0.005	1.08	0.74;1.56	0.694	1.11	0.77;1.61	0.567	1.07	0.74;1.55	0.709	1.05	0.71;1.56	0.815	1.07	0.74;1.55	0.706
Breastfeeding (Yes)	0.65	0.39;1.09	0.103	0.65	0.39;1.09	0.055	0.61	0.37;1.01	0.055	0.63	0.38;1.04	0.529	0.61	0.37;1.02	0.061	0.62	0.37;1.04	0.072	0.61	0.37;1.02	0.061	0.67	0.38;1.17	0.159	0.63	0.37;1.05	0.076
Kin assisting the labor (Yes)	0.80	0.54;1.17	0.245	0.78	0.53;1.14	0.116	0.74	0.51;1.08	0.116	0.76	0.52;1.11	0.071	0.81	0.55;1.19	0.282	0.81	0.55;1.19	0.288	0.77	0.53;1.13	0.189	0.74	0.49;1.12	0.156	0.81	0.55;1.20	0.296
Type of delivery (Natural)	0.88	0.60;1.29	0.502	0.88	0.60;1.29	0.627	0.91	0.62;1.33	0.627	0.89	0.61;1.31	0.152	0.89	0.61;1.31	0.564	0.91	0.62;1.34	0.634	0.89	0.61;1.30	0.546	0.87	0.58;1.31	0.496	0.89	0.60;1.31	0.548
